# Crystal structure of *O*-benzyl-l-tyrosine *N*-carb­oxy anhydride

**DOI:** 10.1107/S2056989017004236

**Published:** 2017-03-21

**Authors:** Aya Inada, Hitoshi Kanazawa

**Affiliations:** a Faculty of Symbiotic Systems Science, Fukushima University, 1 Kanayagawa, Fukushima, 960-1296, Japan

**Keywords:** crystal structure, l-tyrosine, l-amino acid *N*-carb­oxy anhydride, *O*-benzyl-l-tyrosine NCA, solid-state polymerization, hydrogen bonding, C—H⋯π inter­actions

## Abstract

In the title compound, C_17_H_15_NO_4_ (alternative name; *O*-benzyl-l-tyrosine *N*-carb­oxy anhydride), the oxazolidine ring is planer, with an r.m.s. deviation of 0.039 Å. The benz­yloxy and benzyl rings are almost coplanar, making a dihedral angle of 0.078 (10)°, and are inclined to the oxazolidine ring by 59.16 (11) and 58.42 (11)°, respectively. In the crystal, mol­ecules are linked by N—H⋯O and C—H⋯O hydrogen bonds, forming ribbons propagating along [010]. The ribbons are linked by C—H⋯π inter­actions, forming a three-dimensional supra­molecular structure. The oxazolidine rings of adjacent ribbons are arranged into a layer parallel to the *ab* plane. This arrangement is favourable for the polymerization of the compound in the solid state.

## Abstract 2

In the title compound, known also as (*S*)-4-[4-(benz­yloxy)benz­yl]oxazolidine-2,5-dione, the benz­yloxy and benzyl rings are almost coplanar, making a dihedral angle of 0.078 (10)°, and are inclined to the oxazolidine ring by 59.16 (11) and 58.42 (11)°, respectively.

## Chemical context   

*N*-Carb­oxy anhydrides (NCAs) of amino acids are extensively used as monomers for the preparation of high mol­ecular weight polypeptides (Kricheldorf, 2006 ▸). Amino acid NCAs are easily soluble but the resulting polypeptides are not soluble in general organic solvents. Only a few amino acid ester NCAs such as γ-benzyl-l-glutamate NCA (BLG NCA) and β-benzyl-l-aspartate NCA (BLA NCA) are polymerized in solutions, because the resulting polypeptides are soluble in them. On the other hand, we found that every amino acid NCA crystal is polymerized in the solid state in hexane by the initiation of amines. We studied the polymerization of BLA NCA (Kanazawa & Sato, 1996 ▸) and β-benzyl-dl-aspartate NCA (BDLA NCA) initiated by a primary amine in the solution and solid states, and we determined the crystal structure of BLA NCA (Kanazawa & Magoshi, 2003 ▸) and BDLA NCA (Kanazawa & Inada, 2017 ▸) to consider their high reactivity in the solid state. In addition, we prepared single crystals of the title compound, *O*-benzyl-l-tyrosine (OBLT NCA) in hexa­ne–ethyl acetate mixture. The polymerization of OBLT NCA is initiated by butyl amine initiator in dioxane or aceto­nitrile solutions. However, the polymerization rate was extremely slow, because the resultant polymer has a poor solubility in these solvents. On the other hand, the polymerization of OBLT NCA initiated by butyl amine was very reactive in the solid state in hexane. High mol­ecular weight poly(OBLT) was obtained only in the solid-state polymer­ization. High mol­ecular weight poly(OBLT) is valuable, because poly(l-tyrosine) is obtained by the hydration of benzyl groups of the polymer. Therefore, it is important to determine the crystal structure to consider the difference in the reactivity in solution and in the solid state.
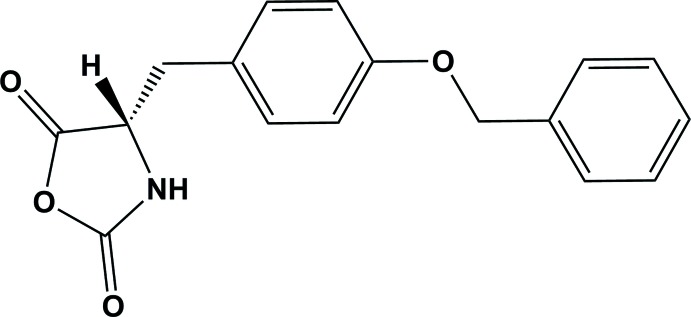


## Structural commentary   

**Figure 1:**
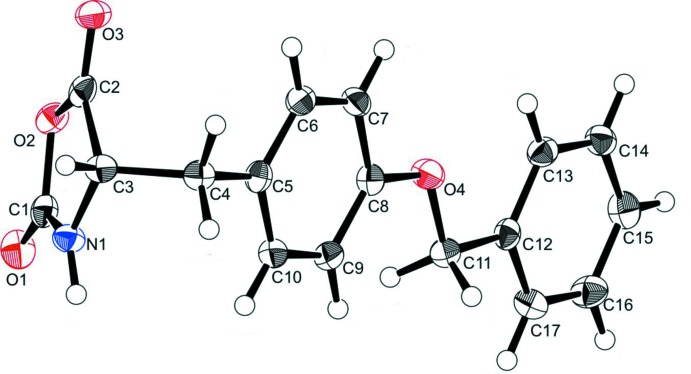
The mol­ecular structure of the title compound, showing the atom labelling and 50% probability displacement ellipsoids.

The mol­ecular structure of the title compound is shown in Fig. 1 ▸. The oxazolidine ring (O2/N1/C1–C3) is planar with an r.m.s. deviation of 0.039 Å, and a maximum deviation of 0.033 (2) Å for atom C3. The side chain has an extended conformation with the C3—C4—C5—C6 and C7—C8—O4—C11 torsion angles being 98.8 (2) and 179.01 (18)°, respectively. Hence, the benz­yloxy (C12–C17) and benzyl (C5–C10) rings are almost coplanar, making a dihedral angle of 0.078 (10)°, and are inclined to the oxazolidine ring by 59.16 (11) and 58.42 (11)°, respectively.

## Supra­molecular features   

**Figure 2:**
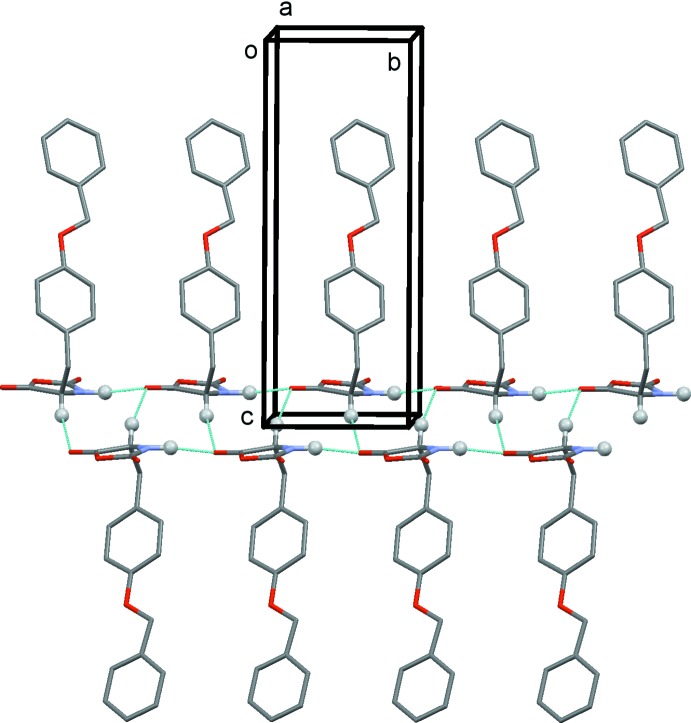
A partial view along the *a* axis of the crystal packing of the title compound. The hydrogen bonds are shown as dashed lines (see Table 1 ▸). For clarity, only H atoms H1 and H3 (grey balls) have been included.

**Figure 3:**
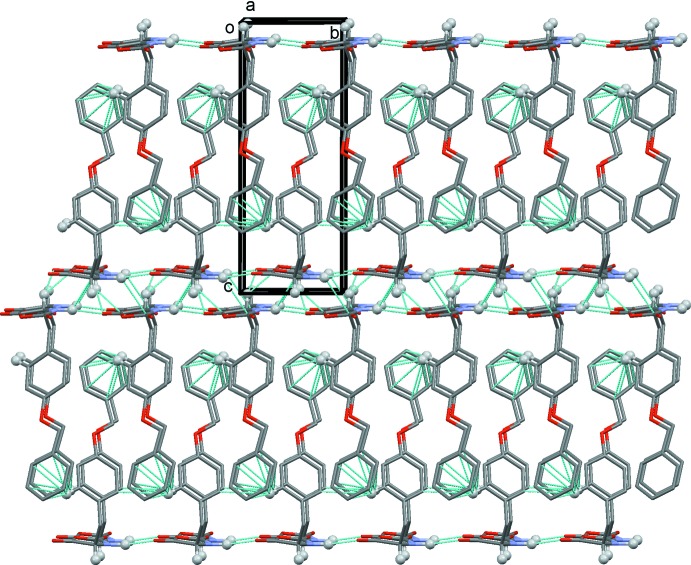
A view along the *a* axis of the crystal packing of the title compound. The hydrogen bonds and C—H⋯π inter­actions are shown as dashed lines (see Table 1 ▸). For clarity, only H atoms H1 and H3 and H6 (grey balls) have been included.

In the crystal, mol­ecules are linked *via* N1—H1⋯O3^i^ and C—H⋯O3^ii^ hydrogen bonds, forming ribbons propagating along the *b-*axis direction (Table 1 ▸ and Fig. 2 ▸). The ribbons are linked by C—H⋯π inter­actions, forming a three-dimensional supra­molecular structure (Table 1 ▸ and Fig. 3 ▸). The five-membered oxazolidine rings are packed in a layer and the –CH_2_C_6_H_4_OCH_2_C_6_H_5_ side chains are packed in another layer; the two different layers stack alternately. This sandwich structure is one of the important requirements for high reactivity in the solid state, because the five-membered rings can react with each other in the layer.

## Database survey   

A search of the Cambridge Structural Database (Version 5.37, update May 2016; Groom *et al.*, 2016 ▸) revealed the presence of 15 hits for 4-methyl­ene-oxazolidine-2,5-dione or 4-ethyl-4-methyl­ene-oxazolidine-2,5-dione derivatives. A number of these compounds involve amino acid side chains (amino acid NCAs). There are four compounds in which a benzyl group side chain is bonded to atom C4 in the oxazolidine-2,5-dione ring, *viz N*-carb­oxy-l-phenyl­alanine anhydride (KIXSUF; Kanazawa, 2000 ▸), *N*-carb­oxy-dl-phenyl­alanine anhydride (RESSUD; Kanazawa *et al.*, 1997 ▸), *N*-carb­oxy-(*R*)-phenyl­alanine anhydride 3-(2-thien­yl) alanine-*N*-carb­oxy­anhydride (SAPYEO; Nery *et al.*,2005 ▸) and *C* ^α^-ethyl-(*S*)-phenyl­alanine-*N*-carb­oxy­anhydride (ZATWEW; Crisma *et al.*,1995 ▸). In these compounds, the dihedral angles between oxazolidine-2,5-dione ring mean plane and the benzene ring are very similar, *viz* 58.42 (11)° in the title compound, 59.34 (15)° in KIXSUF, 55.8 (2) and 54.7 (2)° in RESSUD, 51.7 (7), 50.6 (7)° in SAPYEO and 58.8 (7)° in ZATWEW. Inter­molecular hydrogen bonds are formed between the imino group and the carbonyl O atom in position 2 of the oxazolidine ring in the title compound and in ZATWEW. On the other hand, they are formed between the imino group and the carbonyl O atom at position 5 of the oxazolidine ring in KIXSUF and RESSUD.

## Synthesis and crystallization   

Reagent-grade *O*-benzyl-l-tyrosine (OBLT) (Product Code B3210; Tokyo Kasei Co. Ltd.) was used as received. The title compound was synthesized by the reaction of OBLT with triphosgene in tetra­hydro­furan, as reported previously for the synthesis of BLA NCA (Kanazawa & Magoshi, 2003 ▸). The reaction product was recrystallized slowly in a mixture of ethyl acetate and hexane (1:50 *v*/*v*), avoiding moisture contamination, and gave colourless needle-shaped crystals.

## Refinement   

Crystal data, data collection and structure refinement details are summarized in Table 2 ▸. The N-bound H atom was located in a difference-Fourier map and refined with a distance restraint of N—H = 0.88 (4) Å, with *U*_iso_(H) = 1.14*U*_eq_(N). C-bound H atoms were positioned geometrically and treated as riding: C—H = 0.95–1.00 Å with *U*_iso_(H) = 1.2*U*_eq_(C).

## Supplementary Material

Crystal structure: contains datablock(s) I, Global. DOI: 10.1107/S2056989017004236/su5359sup1.cif

Structure factors: contains datablock(s) I. DOI: 10.1107/S2056989017004236/su5359Isup2.hkl

Supporting information file. DOI: 10.1107/S2056989017004236/su5359Isup3.cml

CCDC reference: 1538344

Additional supporting information:  crystallographic information; 3D view; checkCIF report

## Figures and Tables

**Table 1:** Hydrogen-bond geometry (Å, °) *Cg* is the centroid of the C12–C17 benz­yloxy ring.

*D*—H⋯*A*	*D*—H	H⋯*A*	*D*⋯*A*	*D*—H⋯*A*
N1—H1⋯O3^i^	0.88 (3)	2.09 (3)	2.885 (2)	150 (2)
C3—H3⋯O3^ii^	1.00	2.50	3.410 (3)	151
C6—H6⋯*Cg* ^iii^	0.95	2.89	3.546 (3)	127

Symmetry codes: (i) 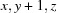; (ii) 
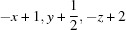
; (iii) 
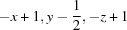
.

**Table 2:** Experimental details

Crystal data	
Chemical formula	C_17_H_15_NO_4_
*M*_r_	297.30
Crystal system, space group	Monoclinic, *P*2_1_
Temperature (K)	123
*a*, *b*, *c* (Å)	7.7388 (5), 5.9128 (4), 15.7769 (10)
β (°)	96.390 (2)
*V* (Å^3^)	717.43 (8)
*Z*	2
Radiation type	Mo *K*α
μ (mm^−1^)	0.10
Crystal size (mm)	0.26 × 0.13 × 0.10

Data collection	
Diffractometer	Rigaku R-AXIS RAPID
Absorption correction	Multi-scan (*ABSCOR*; Higashi, 1995 ▸)
*T*_min_, *T*_max_	0.975, 0.990
No. of measured, independent and observed [*I* > 2σ(*I*)] reflections	6593, 1635, 1444
*R*_int_	0.034
(sin θ/λ)_max_ (Å^−1^)	0.628

Refinement	
*R*[*F* ^2^ > 2σ(*F* ^2^)], *wR*(*F* ^2^), *S*	0.031, 0.068, 1.10
No. of reflections	1635
No. of parameters	202
No. of restraints	1
H-atom treatment	H atoms treated by a mixture of independent and constrained refinement
Δρ_max_, Δρ_min_ (e Å^−3^)	0.20, −0.18

Computer programs: *RAPID-AUTO* (Rigaku, 2009 ▸), *SHELXS97* and *SHELXL97* (Sheldrick, 2008 ▸), *CrystalStructure* (Rigaku, 2009 ▸) and *Mercury* (Macrae *et al.*, 2008 ▸).

## supplementary crystallographic information

### Crystal data

**Table:**

C_17_H_15_NO_4_	*F*(000) = 312
*M**_r_* = 297.30	*D*_x_ = 1.376 Mg m^−^^3^
Monoclinic, *P*2_1_	Mo *K*α radiation, λ = 0.71075 Å
Hall symbol: P 2yb	Cell parameters from 7077 reflections
*a* = 7.7388 (5) Å	θ = 3.5–27.4°
*b* = 5.9128 (4) Å	µ = 0.10 mm^−^^1^
*c* = 15.7769 (10) Å	*T* = 123 K
β = 96.390 (2)°	Needle, colourless
*V* = 717.43 (8) Å^3^	0.26 × 0.13 × 0.10 mm
*Z* = 2	

### Data collection

**Table:**

Rigaku R-AXIS RAPID diffractometer	1635 independent reflections
Radiation source: fine-focus sealed tube	1444 reflections with *I* > 2σ(*I*)
Graphite monochromator	*R*_int_ = 0.034
Detector resolution: 10.0 pixels mm^-1^	θ_max_ = 26.5°, θ_min_ = 3.5°
ω–scan	*h* = −9→9
Absorption correction: multi-scan (ABSCOR; Higashi, 1995)	*k* = −7→7
*T*_min_ = 0.975, *T*_max_ = 0.990	*l* = −19→19
6593 measured reflections	

### Refinement

**Table:**

Refinement on *F*^2^	Primary atom site location: structure-invariant direct methods
Least-squares matrix: full	Secondary atom site location: difference Fourier map
*R*[*F*^2^ > 2σ(*F*^2^)] = 0.031	Hydrogen site location: inferred from neighbouring sites
*wR*(*F*^2^) = 0.068	H atoms treated by a mixture of independent and constrained refinement
*S* = 1.10	*w* = 1/[σ^2^(*F*_o_^2^) + (0.0302*P*)^2^ + 0.1082*P*] where *P* = (*F*_o_^2^ + 2*F*_c_^2^)/3
1635 reflections	(Δ/σ)_max_ < 0.001
202 parameters	Δρ_max_ = 0.20 e Å^−^^3^
1 restraint	Δρ_min_ = −0.18 e Å^−^^3^

### Special details

**Table:**

Geometry. All esds (except the esd in the dihedral angle between two l.s. planes) are estimated using the full covariance matrix. The cell esds are taken into account individually in the estimation of esds in distances, angles and torsion angles; correlations between esds in cell parameters are only used when they are defined by crystal symmetry. An approximate (isotropic) treatment of cell esds is used for estimating esds involving l.s. planes.
Refinement. Refinement of F^2^ against ALL reflections. The weighted R-factor wR and goodness of fit S are based on F^2^, conventional R-factors R are based on F, with F set to zero for negative F^2^. The threshold expression of F^2^ > 2sigma(F^2^) is used only for calculating R-factors(gt) etc. and is not relevant to the choice of reflections for refinement. R-factors based on F^2^ are statistically about twice as large as those based on F, and R- factors based on ALL data will be even larger.

### Fractional atomic coordinates and isotropic or equivalent isotropic displacement parameters (Å^2^)

**Table:**

	*x*	*y*	*z*	*U*_iso_*/*U*_eq_	
O1	1.08406 (18)	0.6360 (3)	0.91435 (9)	0.0310 (4)	
O2	0.89115 (18)	0.3443 (3)	0.91282 (8)	0.0238 (3)	
O3	0.6500 (2)	0.1393 (3)	0.92135 (9)	0.0290 (4)	
O4	0.70846 (18)	0.5324 (3)	0.52871 (9)	0.0265 (4)	
N1	0.7966 (2)	0.6908 (3)	0.93804 (11)	0.0229 (4)	
H1	0.792 (3)	0.840 (6)	0.9383 (16)	0.026*	
C1	0.9379 (3)	0.5765 (4)	0.92122 (12)	0.0234 (5)	
C2	0.7201 (3)	0.3211 (4)	0.92381 (12)	0.0221 (5)	
C3	0.6426 (3)	0.5499 (4)	0.93569 (13)	0.0213 (4)	
H3	0.5941	0.5568	0.9918	0.026*	
C4	0.4993 (2)	0.6067 (4)	0.86274 (12)	0.0222 (5)	
H4A	0.4003	0.5022	0.8659	0.027*	
H4B	0.4570	0.7622	0.8712	0.027*	
C5	0.5591 (2)	0.5901 (4)	0.77459 (12)	0.0212 (5)	
C6	0.5233 (3)	0.3969 (4)	0.72446 (13)	0.0227 (5)	
H6	0.4620	0.2743	0.7461	0.027*	
C7	0.5764 (3)	0.3826 (4)	0.64309 (13)	0.0229 (5)	
H7	0.5524	0.2500	0.6098	0.027*	
C8	0.6648 (2)	0.5620 (4)	0.61046 (13)	0.0218 (4)	
C9	0.7028 (3)	0.7545 (4)	0.65934 (13)	0.0230 (5)	
H9	0.7644	0.8766	0.6376	0.028*	
C10	0.6491 (3)	0.7661 (4)	0.74112 (13)	0.0233 (5)	
H10	0.6750	0.8978	0.7746	0.028*	
C11	0.7964 (3)	0.7139 (4)	0.49268 (12)	0.0236 (5)	
H11A	0.9058	0.7481	0.5292	0.028*	
H11B	0.7226	0.8510	0.4896	0.028*	
C12	0.8365 (2)	0.6502 (4)	0.40454 (13)	0.0215 (4)	
C13	0.7879 (3)	0.4450 (4)	0.36549 (13)	0.0231 (5)	
H13	0.7276	0.3355	0.3950	0.028*	
C14	0.8277 (3)	0.4004 (4)	0.28317 (13)	0.0245 (5)	
H14	0.7950	0.2597	0.2571	0.029*	
C15	0.9141 (3)	0.5582 (4)	0.23910 (13)	0.0281 (5)	
H15	0.9394	0.5271	0.1827	0.034*	
C16	0.9636 (3)	0.7619 (4)	0.27748 (14)	0.0293 (5)	
H16	1.0239	0.8706	0.2476	0.035*	
C17	0.9253 (3)	0.8080 (4)	0.35980 (13)	0.0265 (5)	
H17	0.9598	0.9482	0.3858	0.032*	

### Atomic displacement parameters (Å^2^)

**Table:**

	*U*^11^	*U*^22^	*U*^33^	*U*^12^	*U*^13^	*U*^23^
O1	0.0254 (8)	0.0395 (10)	0.0284 (7)	−0.0028 (8)	0.0048 (6)	0.0012 (7)
O2	0.0264 (7)	0.0216 (8)	0.0242 (7)	0.0054 (7)	0.0067 (6)	0.0018 (7)
O3	0.0374 (9)	0.0201 (8)	0.0295 (8)	−0.0022 (7)	0.0040 (7)	0.0011 (7)
O4	0.0333 (8)	0.0254 (9)	0.0214 (7)	−0.0067 (7)	0.0060 (6)	−0.0013 (6)
N1	0.0234 (9)	0.0190 (10)	0.0264 (8)	0.0002 (8)	0.0030 (7)	−0.0026 (8)
C1	0.0263 (11)	0.0272 (12)	0.0164 (9)	0.0002 (10)	0.0020 (8)	0.0030 (9)
C2	0.0287 (11)	0.0244 (12)	0.0138 (9)	0.0034 (10)	0.0051 (8)	0.0031 (8)
C3	0.0249 (10)	0.0194 (11)	0.0208 (9)	−0.0005 (9)	0.0084 (8)	−0.0008 (9)
C4	0.0198 (9)	0.0218 (12)	0.0254 (10)	0.0014 (9)	0.0044 (8)	−0.0006 (9)
C5	0.0156 (9)	0.0241 (13)	0.0232 (9)	0.0043 (9)	−0.0002 (8)	0.0016 (9)
C6	0.0189 (10)	0.0225 (12)	0.0264 (10)	−0.0013 (9)	0.0008 (8)	0.0031 (9)
C7	0.0209 (10)	0.0209 (12)	0.0258 (10)	−0.0011 (9)	−0.0024 (8)	−0.0025 (9)
C8	0.0204 (10)	0.0243 (11)	0.0203 (9)	0.0012 (10)	0.0012 (8)	0.0001 (9)
C9	0.0243 (10)	0.0212 (11)	0.0232 (10)	−0.0020 (9)	0.0012 (8)	0.0033 (9)
C10	0.0243 (11)	0.0207 (12)	0.0242 (10)	0.0004 (9)	−0.0009 (8)	−0.0004 (9)
C11	0.0250 (10)	0.0223 (12)	0.0234 (10)	−0.0022 (9)	0.0020 (8)	0.0017 (9)
C12	0.0172 (9)	0.0249 (11)	0.0217 (9)	0.0013 (9)	−0.0006 (8)	0.0028 (9)
C13	0.0212 (11)	0.0216 (11)	0.0265 (10)	0.0009 (9)	0.0021 (8)	0.0028 (9)
C14	0.0223 (11)	0.0228 (12)	0.0277 (10)	0.0006 (9)	0.0001 (9)	−0.0018 (9)
C15	0.0263 (11)	0.0344 (13)	0.0241 (10)	0.0029 (11)	0.0045 (8)	−0.0038 (10)
C16	0.0332 (12)	0.0275 (13)	0.0287 (11)	−0.0040 (10)	0.0105 (9)	0.0038 (10)
C17	0.0264 (11)	0.0240 (13)	0.0290 (11)	−0.0039 (10)	0.0024 (9)	−0.0024 (10)

### Geometric parameters (Å, º)

**Table:**

O1—C1	1.201 (2)	C7—H7	0.9500
O2—C2	1.361 (2)	C8—C9	1.388 (3)
O2—C1	1.422 (3)	C9—C10	1.400 (3)
O3—C2	1.202 (3)	C9—H9	0.9500
O4—C8	1.380 (2)	C10—H10	0.9500
O4—C11	1.423 (3)	C11—C12	1.506 (3)
N1—C1	1.337 (3)	C11—H11A	0.9900
N1—C3	1.451 (3)	C11—H11B	0.9900
N1—H1	0.88 (3)	C12—C13	1.393 (3)
C2—C3	1.500 (3)	C12—C17	1.396 (3)
C3—C4	1.544 (3)	C13—C14	1.393 (3)
C3—H3	1.0000	C13—H13	0.9500
C4—C5	1.517 (3)	C14—C15	1.380 (3)
C4—H4A	0.9900	C14—H14	0.9500
C4—H4B	0.9900	C15—C16	1.383 (3)
C5—C10	1.389 (3)	C15—H15	0.9500
C5—C6	1.399 (3)	C16—C17	1.391 (3)
C6—C7	1.394 (3)	C16—H16	0.9500
C6—H6	0.9500	C17—H17	0.9500
C7—C8	1.391 (3)		
			
C2—O2—C1	109.05 (16)	O4—C8—C7	115.5 (2)
C8—O4—C11	117.10 (17)	C9—C8—C7	120.15 (18)
C1—N1—C3	113.10 (19)	C8—C9—C10	119.05 (19)
C1—N1—H1	122.7 (16)	C8—C9—H9	120.5
C3—N1—H1	122.7 (16)	C10—C9—H9	120.5
O1—C1—N1	132.0 (2)	C5—C10—C9	121.8 (2)
O1—C1—O2	120.44 (19)	C5—C10—H10	119.1
N1—C1—O2	107.53 (17)	C9—C10—H10	119.1
O3—C2—O2	121.8 (2)	O4—C11—C12	109.70 (18)
O3—C2—C3	128.69 (18)	O4—C11—H11A	109.7
O2—C2—C3	109.44 (18)	C12—C11—H11A	109.7
N1—C3—C2	100.53 (16)	O4—C11—H11B	109.7
N1—C3—C4	114.45 (17)	C12—C11—H11B	109.7
C2—C3—C4	111.52 (18)	H11A—C11—H11B	108.2
N1—C3—H3	110.0	C13—C12—C17	118.84 (18)
C2—C3—H3	110.0	C13—C12—C11	123.57 (19)
C4—C3—H3	110.0	C17—C12—C11	117.6 (2)
C5—C4—C3	113.72 (15)	C14—C13—C12	120.03 (19)
C5—C4—H4A	108.8	C14—C13—H13	120.0
C3—C4—H4A	108.8	C12—C13—H13	120.0
C5—C4—H4B	108.8	C15—C14—C13	120.8 (2)
C3—C4—H4B	108.8	C15—C14—H14	119.6
H4A—C4—H4B	107.7	C13—C14—H14	119.6
C10—C5—C6	118.23 (17)	C14—C15—C16	119.63 (19)
C10—C5—C4	121.1 (2)	C14—C15—H15	120.2
C6—C5—C4	120.62 (18)	C16—C15—H15	120.2
C7—C6—C5	120.66 (19)	C15—C16—C17	120.2 (2)
C7—C6—H6	119.7	C15—C16—H16	119.9
C5—C6—H6	119.7	C17—C16—H16	119.9
C8—C7—C6	120.1 (2)	C16—C17—C12	120.6 (2)
C8—C7—H7	119.9	C16—C17—H17	119.7
C6—C7—H7	119.9	C12—C17—H17	119.7
O4—C8—C9	124.34 (19)		
			
C3—N1—C1—O1	176.8 (2)	C11—O4—C8—C7	179.01 (18)
C3—N1—C1—O2	−4.5 (2)	C6—C7—C8—O4	−178.74 (18)
C2—O2—C1—O1	179.56 (17)	C6—C7—C8—C9	1.1 (3)
C2—O2—C1—N1	0.7 (2)	O4—C8—C9—C10	178.99 (19)
C1—O2—C2—O3	−178.53 (18)	C7—C8—C9—C10	−0.8 (3)
C1—O2—C2—C3	3.2 (2)	C6—C5—C10—C9	0.4 (3)
C1—N1—C3—C2	6.0 (2)	C4—C5—C10—C9	−179.00 (19)
C1—N1—C3—C4	−113.59 (19)	C8—C9—C10—C5	0.1 (3)
O3—C2—C3—N1	176.5 (2)	C8—O4—C11—C12	178.88 (17)
O2—C2—C3—N1	−5.4 (2)	O4—C11—C12—C13	1.7 (3)
O3—C2—C3—C4	−61.8 (3)	O4—C11—C12—C17	−179.17 (18)
O2—C2—C3—C4	116.34 (17)	C17—C12—C13—C14	−0.1 (3)
N1—C3—C4—C5	58.2 (2)	C11—C12—C13—C14	179.01 (19)
C2—C3—C4—C5	−55.1 (2)	C12—C13—C14—C15	−0.5 (3)
C3—C4—C5—C10	−81.9 (2)	C13—C14—C15—C16	0.8 (3)
C3—C4—C5—C6	98.8 (2)	C14—C15—C16—C17	−0.5 (3)
C10—C5—C6—C7	−0.1 (3)	C15—C16—C17—C12	−0.1 (3)
C4—C5—C6—C7	179.27 (18)	C13—C12—C17—C16	0.4 (3)
C5—C6—C7—C8	−0.6 (3)	C11—C12—C17—C16	−178.8 (2)
C11—O4—C8—C9	−0.8 (3)		

### Hydrogen-bond geometry (Å, º)

*Cg* is the centroid of the C12–C17 benzyloxy ring.

**Table:**

*D*—H···*A*	*D*—H	H···*A*	*D*···*A*	*D*—H···*A*
N1—H1···O3^i^	0.88 (3)	2.09 (3)	2.885 (2)	150 (2)
C3—H3···O3^ii^	1.00	2.50	3.410 (3)	151
C6—H6···*Cg*^iii^	0.95	2.89	3.546 (3)	127

Symmetry codes: (i) *x*, *y*+1, *z*; (ii) −*x*+1, *y*+1/2, −*z*+2; (iii) −*x*+1, *y*−1/2, −*z*+1.

## References

[bb1] Crisma, M., Valle, G., Formaggio, F., Toniolo, C. & Kamphui, J. (1995). *Z. Kristallogr.* **210**, 634–635.

[bb13] Groom, C. R., Bruno, I. J., Lightfoot, M. P. & Ward, S. C. (2016). *Acta Cryst* B**72**, 171–179. 10.1107/S2052520616003954 PMC4822653 27048719

[bb2] Higashi, T. (1995). *ABSCOR*. Rigaku Corporation, Tokyo, Japan.

[bb3] Kanazawa, H. (2000). *Acta Cryst.* C**56**, 469–470. 10.1107/S0108270100000330 10815213

[bb4] Kanazawa, H. & Inada, A. (2017). *Acta Cryst.* E**73**, 445–447. 10.1107/S2056989017003024 PMC5347073 28316828

[bb5] Kanazawa, H. & Magoshi, J. (2003). *Acta Cryst.* C**59**, o159–o161. 10.1107/s0108270103002567 12711795

[bb6] Kanazawa, H. & Sato, Y. (1996). *Science Reports, Fukushima University.* **59**, 13–17.

[bb7] Kanazawa, H., Uekusa, H. & Ohashi, Y. (1997). *Acta Cryst.* C**53**, 1154–1156.

[bb8] Kricheldorf, H. R. (2006). *Angew. Chem. Int. Ed.* **45**, 5752–5784. 10.1002/anie.200600693 16948174

[bb9] Macrae, C. F., Bruno, I. J., Chisholm, J. A., Edgington, P. R., McCabe, P., Pidcock, E., Rodriguez-Monge, L., Taylor, R., van de Streek, J. & Wood, P. A. (2008). *J. Appl. Cryst.* **41**, 466–470.

[bb10] Nery, J. G., Bolbach, G., Weissbuch, G. & Lahav, M. (2005). *Chem. Eur. J.* **11**, 3039–3048. 10.1002/chem.200401097 15770708

[bb11] Rigaku (2009). *RAPID-AUTO* and *CrystalStructure*, Rigaku Corporation. Tokyo, Japan.

[bb12] Sheldrick, G. M. (2008). *Acta Cryst.* A**64**, 112–122. 10.1107/S0108767307043930 18156677

